# Equilibrative Nucleoside Transporters Mediate the Import of Nicotinamide Riboside and Nicotinic Acid Riboside into Human Cells

**DOI:** 10.3390/ijms22031391

**Published:** 2021-01-30

**Authors:** Andrey Kropotov, Veronika Kulikova, Kirill Nerinovski, Alexander Yakimov, Maria Svetlova, Ljudmila Solovjeva, Julia Sudnitsyna, Marie E. Migaud, Mikhail Khodorkovskiy, Mathias Ziegler, Andrey Nikiforov

**Affiliations:** 1 Institute of Cytology, Russian Academy of Sciences, St. Petersburg 194064, Russia; a.kropotov@gmail.com (A.K.); veronika.a.kulikova@gmail.com (V.K.); svetlma@mail.ru (M.S.); mila.solovjeva@gmail.com (L.S.); khodorkovskii@gmail.com (M.K.); 2 Sechenov Institute of Evolutionary Physiology and Biochemistry, Russian Academy of Sciences, St. Petersburg 194223, Russia; julia.sudnitsyna@gmail.com; 3 Department of Nuclear Physics Research Methods, St. Petersburg State University, St. Petersburg 199034, Russia; nerinovski@yandex.ru; 4 Peter the Great St. Petersburg Polytechnic University, St. Petersburg 195251, Russia; yaleks@gmail.com; 5 Mitchell Cancer Institute, University of South Alabama, Mobile, AL 36604, USA; mmigaud@health.southalabama.edu; 6 Department of Biomedicine, University of Bergen, 5020 Bergen, Norway; Mathias.Ziegler@uib.no

**Keywords:** NAD^+^ metabolism, nicotinamide riboside, nicotinic acid riboside, equilibrative nucleoside transporters, concentrative nucleoside transporters, human cells

## Abstract

Nicotinamide riboside (NR), a new form of vitamin B3, is an effective precursor of nicotinamide adenine dinucleotide (NAD^+^) in human and animal cells. The introduction of NR into the body effectively increases the level of intracellular NAD^+^ and thereby restores physiological functions that are weakened or lost in experimental models of aging and various pathologies. Despite the active use of NR in applied biomedicine, the mechanism of its transport into mammalian cells is currently not understood. In this study, we used overexpression of proteins in HEK293 cells, and metabolite detection by NMR, to show that extracellular NR can be imported into cells by members of the equilibrative nucleoside transporter (ENT) family ENT1, ENT2, and ENT4. After being imported into cells, NR is readily metabolized resulting in Nam generation. Moreover, the same ENT-dependent mechanism can be used to import the deamidated form of NR, nicotinic acid riboside (NAR). However, NAR uptake into HEK293 cells required the stimulation of its active utilization in the cytosol such as phosphorylation by NR kinase. On the other hand, we did not detect any NR uptake mediated by the concentrative nucleoside transporters (CNT) CNT1, CNT2, or CNT3, while overexpression of CNT3, but not CNT1 or CNT2, moderately stimulated NAR utilization by HEK293 cells.

## 1. Introduction

In mammalian cells and tissues, the coenzyme NAD^+^ is rapidly turned over [1] because it is consumed by several families of regulatory proteins, such as sirtuin protein deacetylases (SIRTs), poly(ADP-ribose) polymerases (PARPs), and the ADP-ribosyl cyclases CD38 and SARM1. These proteins control many vital processes [2,3,4], and they all cleave the dinucleotide into ADP-ribose and nicotinamide (Nam). Nam, a form of vitamin B3, is largely recycled into NAD^+^ synthesis through Nam phosphoribosyltransferase (NamPRT) forming nicotinamide mononucleotide (NMN). In recent years, alternative NAD^+^ precursors have been studied that have the potential to efficiently replenish cellular pools of the coenzyme. Among them, the ribosylated form of Nam, NR, has emerged as a promising candidate. NR is converted to NMN by NR kinases [5]. Recently, it has been shown that the reduced form of NR, NRH, can enter NAD^+^ biosynthesis via phosphorylation by adenosine kinase [6,7]. The deamidated forms of Nam and NR, nicotinic acid (NA) and its ribosylated form (NAR) are also utilized as NAD^+^ precursors. While NA has been well known as another form of vitamin B3 that is converted to NAD^+^ in the Preiss-Handler pathway, the role of NAR as NAD^+^ precursor still awaits comprehensive characterization. Studying the potential of alternatives to Nam for NAD^+^ biosynthesis is important, because imbalances in NAD^+^ homeostasis are associated with a wide range of pathologies. In particular, a significant drop in the NAD^+^ level is observed with the development of metabolic [8,9] and neurodegenerative [10,11,12] disorders, heart disease [13,14], muscle atrophy [15], and renal dysfunction [16]. Moreover, during aging, the concentration of NAD^+^ decreases in many tissues in rodents [9,17,18,19] and humans [20,21].

The introduction of various forms of vitamin B3 into the body effectively increases the level of intracellular NAD^+^ and thereby restores physiological functions that are weakened or lost in experimental models of aging and various pathologies [22,23]. NR has been used in a number of investigations to boost cellular NAD^+^ levels. While in some studies NR supplementation did not have beneficial effects [24,25,26], in others NR can efficiently enhance NAD^+^ levels thereby improving conditions in mouse models of diseases such as Alzheimer’s Disease [27], noise-induced hearing loss [12], high-fat diet-induced obesity [28], and type 2 diabetes [8]. NR increases the lifespan of mice [29], while its administration to humans is non-toxic and leads to an increase in the level of NAD^+^ in the blood [30,31]. Currently, active clinical trials of NR are underway with the participation of healthy volunteers, patients with prediabetes, obesity, aged individuals, as well as patients with moderate cognitive impairment.

Despite such an active use of NR in applied biomedicine, the mechanisms of its uptake into human cells, a key step in its physiological effect, have not been elucidated. Likewise, we and others have previously demonstrated that NAR can act as efficient NAD^+^ biosynthetic precursor in cultures of mammalian cells [32,33,34]. However, how NAR enters the cells remains unknown.

In humans, purine and pyrimidine nucleosides enter cells through carriers belonging to two families of transmembrane proteins, SLC29 and SLC28. The SLC29 family includes equilibrative nucleoside transporters (ENTs) that allow nucleoside diffusion across the plasma membrane and have broad substrate specificity. In contrast, SLC28 proteins are highly specific concentrating nucleoside transporters (CNTs) that couple the transport of Na^+^ (and in some cases H^+^) with nucleoside translocation. The SLC29 family of transporters includes four proteins (ENT1–4), while the SLC28 family consists of three members (CNT1–3) [35,36].

Based on observations obtained using pharmacological inhibition of ENTs in cultured human cells, we previously suggested that members of this family can participate in the transport of NR across the plasma membrane [37]. In this study, we tested whether members of the ENT as well as CNT families could mediate the import of NR into cells. Using overexpression of the corresponding proteins in human cells and metabolite detection by NMR, we identified the carriers involved in NR uptake. In addition, we have characterized the mechanism of import into cells of the less-studied nucleoside NAR.

## 2. Results

### 2.1. ENT Inhibition Suppresses NR Utilization by HEK293 Cells for NAD^+^ Biosynthesis

**Figure 1:**
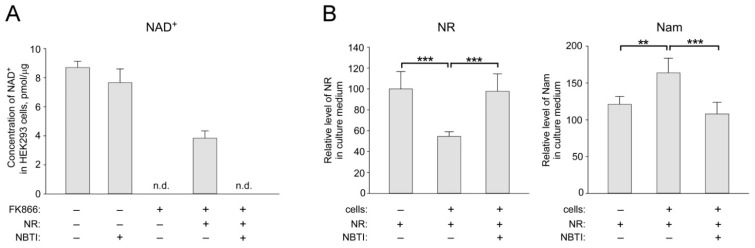
The effect of equilibrative nucleoside transporter (ENT) inhibition on NR utilization by HEK293 cells for NAD^+^ biosynthesis. HEK293 cells were cultivated in Dulbecco’s modified Eagle’s medium (DMEM) containing Nam, supplemented with 10% fetal bovine serum (FBS). Cells were treated with nicotinamide riboside (NR) (100 µM) and inhibitor of equilibrative nucleoside transporters S-(4-nitrobenzyl)-6-thioinosine (NBTI) (10 µM) as indicated. To inhibit NAD^+^ synthesis from Nam, cells were treated with FK866 (2 µM) (**A**). Twenty-four hours after the treatment, cell extracts (**A**) and culture medium (**B**) were analyzed by quantitative NMR spectroscopy. (**A**) The concentration of intracellular NAD^+^ is expressed in picomoles per microgram of total protein in cell extract. Data are presented as mean ± S.D. n.d.—not detected. (**B**) Relative levels of NR and Nam in culture medium are presented. Amount of NR in control culture medium incubated without cells was taken as 100. Data are presented as mean ± S.D (*n* = 3). Statistical analysis of differences between the groups was carried out by one-way ANOVA with post hoc comparisons using Tukey test. ** indicates statistical significance at *p* < 0.01, *** indicates statistical significance at *p* < 0.001.

First, we confirmed that pharmacological inhibition of ENTs suppresses NAD^+^ synthesis from NR in cultured HEK293 cells. Since the standard culture medium contains only Nam as NAD^+^ precursor, the addition of FK866, an inhibitor of Nam phosphoribosyltransferase, NamPRT [38], rapidly depletes intracellular NAD^+^ stores (Figure 1A and Figure S1A). 

When both NR (100 μM) and FK866 were present, the NAD^+^ level in the cells was partially restored to about 44% of the control level (Figure 1A). However, when adding NBTI, an inhibitor of ENTs [39] to the medium, cells lost the ability to synthesize NAD^+^ from extracellular NR. Moreover, NBTI treatment did not affect the ability of the cells to synthesize NAD^+^ from Nam in the control sample (Figure 1A). Similar results were obtained using HeLa cells (Figure S1B). ^1^H-NMR analysis of conditioned medium obtained from HEK293 cells cultured in the presence of 100 μM NR revealed intensive utilization of extracellular NR (Figure 1B, left panel and Figure S1C). After 24 h of incubation, the amount of NR in the nutrient medium dropped to 55%. At the same time, the Nam concentration in the medium increased proportionally (Figure 1B, right panel). Neither a drop in the NR concentration nor an increase in the Nam level in the culture medium was observed when NBTI was added to the cells in addition to NR (Figure 1B). Thus, NR is imported into HEK293 cells by NBTI-sensitive transporter(s) and eventually converted to Nam. Nam is released from the cells into the culture medium. These results are in line and extend our previous observations using different experimental conditions [40].

### 2.2. ENT1, ENT2 and ENT4 Mediate NR Uptake into Human Cells

**Figure 2:**
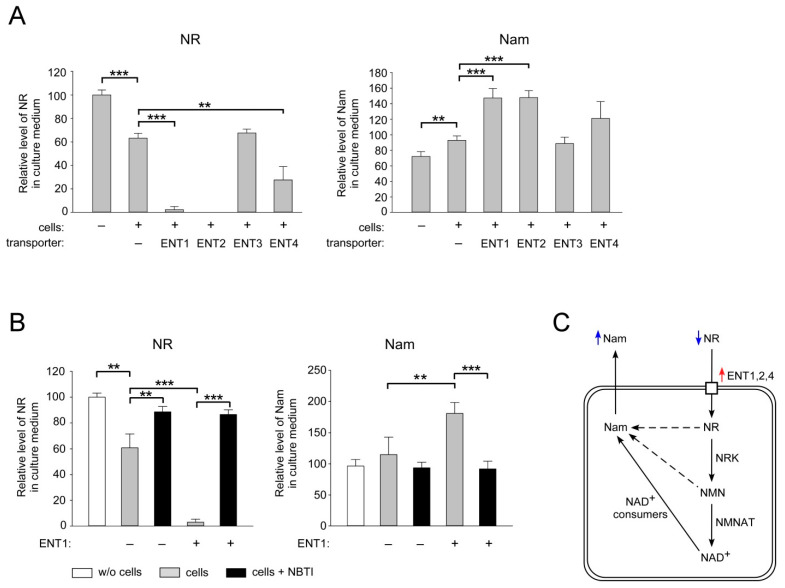
Overexpression of ENT1, ENT2 and ENT4 stimulates NR uptake into HEK293 cells. (**A**,**B**) HEK293 cells cultivated in DMEM were transiently transfected with empty vector or with vectors encoding FLAG-tagged ENT1, 2, 3, or 4 as indicated. Twenty-four hours after transfection, cells were treated with NR (100 µM). In (**B**), cells were additionally treated with NBTI (10 µM) as indicated. Twenty-four hours after treatment, culture medium was analyzed by NMR spectroscopy. Relative levels of NR and Nam in culture medium are presented. The amount of NR in control culture media incubated without cells was taken as 100. Data are presented as mean ± S.D (*n* = 3). Statistical analysis of differences between the groups was carried out by one-way ANOVA with post hoc comparisons using Tukey test. ** indicates statistical significance at *p* < 0.01, *** indicates statistical significance at *p* < 0.001. (**C**) Schematic interpretation of the results shown in (**A**,**B**). Blue arrows: changes in extracellular metabolite concentrations; red arrow: overexpression of FLAG-tagged proteins.

Next, we tested whether the proteins of the ENT family mediate NR uptake into human cells. We overexpressed ENTs1–4 endowed with an N-terminal FLAG tag in HEK293 cells and evaluated changes in the rate of utilization of extracellular NR by cells. Unlike other members of the ENT family, the ENT3 protein is localized in intracellular membranes and does not mediate the uptake of extracellular nucleosides [41,42]. Therefore, overexpression of this transporter was used as a negative control. The efficiency of overexpression of the FLAG-fusion proteins was assessed by immunoblotting using antibodies recognizing the FLAG peptide (Figure S2A). Twenty-four hours after transfection, NR was added to the culture medium at a concentration of 100 μM. After another 24 h, the conditioned culture medium was analyzed by NMR spectroscopy. Incubation of HEK293 cells transfected with the empty vector with NR led to a similar decrease in the amount of the nucleoside in the culture medium (Figure 2A, left panel) and to a concomitant increase in extracellular Nam (Figure 2A, right panel), as observed in the experiment with untransfected cells (cf. Figure 1B).

After overexpression of FLAG-tagged ENT1, ENT2, and ENT4, the amount of NR in the medium dramatically decreased compared to samples obtained from mock-transfected cells. Almost all NR was utilized by cells after overexpression of ENT1, while following overexpression of ENT2, remaining NR, if any, in the medium was undetectable. Overexpression of ENT4 stimulated NR consumption less strongly (Figure 2A, left panel). At the same time, in all samples, the amount of Nam increased proportionally (Figure 2A, right panel). As expected, after overexpression of ENT3, the amount of NR and Nam in the culture medium remained at control levels (Figure 2A). It is noteworthy that NBTI very efficiently blocked the enforced NR consumption by ENT1 overexpression (Figure 2B, left panel). Likewise, in the presence of NBTI, no Nam accumulation in the medium was observed (Figure 2B, right panel). Thus, overexpression of ENT1, ENT2, and ENT4 considerably increased NR uptake and consumption by HEK293 cells (Figure 2C). These results indicate that conversion of NR to nicotinamide occurred intra- rather than extracellularly. The formation of Nam can be explained by the fact that NR is metabolized into NAD^+^, which is then converted to Nam by NAD^+^-consuming enzymes. It cannot be ruled out that NR or its phosphorylated form, NMN, are converted to Nam prior to their conversion to NAD^+^. Nam is then released from the cells into the culture medium where it is detected (Figure 2C). These results suggest that members of the ENT family can mediate NR uptake into human cells.

### 2.3. Overexpression of CNT1, CNT2, and CNT3 in Human Cells does not Affect the Import of NR into HEK293 Cells

**Figure 3:**
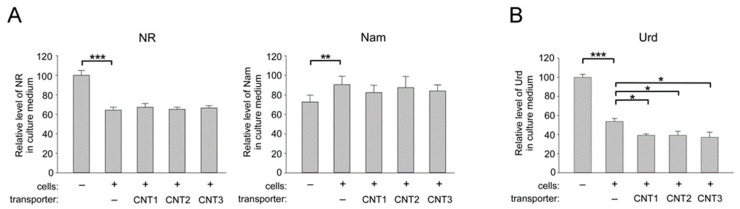
Overexpression of CNT1, CNT2, and CNT3 does not affect the import of NR into HEK293 cells. HEK293 cells cultivated in DMEM were transiently transfected with control (empty) vector or with vectors encoding FLAG-tagged CNT1, 2, or 3 as indicated. Twenty-four hours after transfection, cells were treated with NR (100 µM) (**A**) or with uridine (Urd) (150 µM) (**B**). Twenty-four hours after treatment, culture medium was analyzed by NMR spectroscopy. (**A**) Relative levels of NR and Nam in culture medium are presented. The amount of NR in control culture medium incubated without cells was taken as 100. (**B**) Relative levels of Urd in culture medium are presented. The amount of Urd in control culture medium incubated without cells was taken as 100. Data are presented as mean ± S.D (*n* = 3). Statistical analysis of differences between the groups was carried out by a one-way ANOVA with post hoc comparisons using Tukey test. * indicates statistical significance at *p* < 0.05, ** indicates statistical significance at *p* < 0.01, *** indicates statistical significance at *p* < 0.001.

To establish whether representatives of another family of nucleoside carriers, CNT, might participate in the transport of NR into human cells, FLAG-tagged CNT1, CNT2, or CNT3 were overexpressed in HEK293 cells following transient transfection with the appropriate vectors (Figure S2A). Twenty-four hours after transfection, NR (100 μM) was added to the cells for 24 h. Thereafter, the culture medium was collected and analyzed by NMR. As can be inferred from Figure 3A (left panel) and Figure S2B, none of the overexpressed CNTs affected the efficiency of NR consumption by HEK293 cells.

Consequently, compared to control-transfected cells, no additional accumulation of Nam in the medium was detected (Figure 3A, right panel). As a control, uridine (Urd), a well-described substrate of the CNT family of transporters [35], was tested. After incubation with cells transfected with an empty vector, the amount of Urd in the culture medium dropped to 55% of the level of this nucleoside in the medium incubated without cells (Figure 3B and Figure S3). Overexpression of either CNT1, CNT2, or CNT3 led to a significant decrease in the amount of Urd in the medium compared to samples obtained from mock-transfected cells. Thus, the FLAG-fusion proteins CNT1–3 were functionally active, since their overexpression stimulated Urd uptake into HEK293 cells. Therefore, members of the CNT family do not appear to mediate the transport of NR into human cells.

### 2.4. ENT1, ENT2, and ENT4 Mediate NAR Uptake into Human Cells

**Figure 4:**
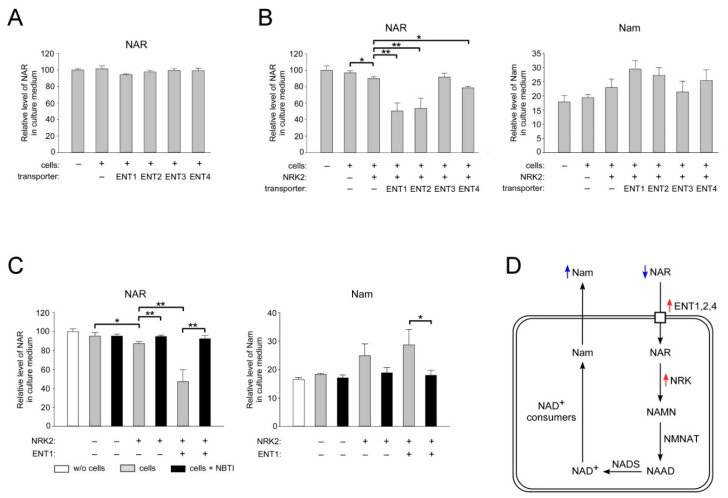
Co-expression of NRK2 and ENT1, ENT2, or ENT4 stimulates NAR uptake into HEK293 cells. HEK293 cells cultivated in DMEM were transiently transfected with vectors encoding FLAG-tagged ENT1, -2, -3, or -4 or control (empty) vector (**A**). Cells were also cotransfected with a vector encoding FLAG-tagged NRK2 as indicated (**B**,**C**). Twenty-four hours after transfection, cells were treated with nicotinic acid riboside NAR (100 µM). In (**C**), cells were additionally treated with NBTI (10 µM) as indicated. Twenty-four hours after treatment, culture medium was analyzed by NMR spectroscopy. Relative levels of NAR and Nam in culture medium are presented. The amount of NAR in control culture media incubated without cells was taken as 100. Data are presented as mean ± S.D (*n* = 3). Statistical analysis of differences between the groups was carried out by one-way ANOVA with post hoc comparisons using Tukey test. * indicates statistical significance at *p* < 0.05, ** indicates statistical significance at *p* < 0.01. (**D**) Schematic interpretation of the results shown in (**A**–**C**). Blue arrows: changes in extracellular metabolite concentrations; red arrows: overexpressions of FLAG-tagged proteins.

Next, we decided to establish whether the ENT-dependent import mechanism into cells also extends to the deamidated nucleoside form of vitamin B3, NAR. We used the same experimental approach as for the study of NR uptake. First, we confirmed that extracellular NAR supports NAD^+^ generation in HEK293 cells (Figure S4). Surprisingly, after 24 h of incubation of HEK293 cells with NAR, the amount of the nucleoside in the culture medium was unchanged, as it remained at the same level as during incubation in medium without cells (Figure 4A, first two columns, and Figure S5).

This observation implies that NAR is not utilized by HEK293 cells as actively as NR under similar conditions (cf. Figure 1B). Moreover, overexpression of ENT1, ENT2 or ENT4 had no effect on the apparent absence of NAR consumption by HEK293 cells (Figure 4A and Figure S5A). Proteins of the ENT family facilitate the diffusion of nucleosides across the plasma membrane. Therefore, the direction and efficiency of transport across the membrane will depend on the difference in the concentration of the metabolite outside the cell and in the cytosol. Since, in the presence of Nam, NAR may not be actively metabolized in the cytosol, an equilibrium may be quickly established between the intra- and extracellular pools of this nucleoside. Therefore, we reasoned that even if the NAR uptake is mediated by ENT1, -2, and -4, we may not detect an effect of their overexpression on the amount of extracellular NAR under the chosen experimental conditions, given that the NAR concentration in the medium is relatively high. To create a concentration gradient of NAR and thereby activate the putative ENT-dependent uptake, we decided to stimulate the “clearance” of NAR from the cytosol by its phosphorylation. HEK293 cells were transfected with a vector encoding FLAG-tagged NRK2. Twenty-four hours after transfection, NAR (100 μM) was added to the cells for 24 h. Next, the culture medium was collected and analyzed by NMR. Indeed, overexpression of NRK2 led to a small, but statistically significant drop in the level of extracellular NAR (Figure 4A, left panel). Importantly, when NRK2 was co-expressed with ENT1, ENT2, or ENT4 proteins, the NAR level in the medium dropped by 48, 46, and 14%, respectively, compared to the decrease obtained from cells expressing NRK2 alone (Figure 4B, left panel). At the same time, when ENT3 was co-expressed with NRK2, the amount of NAR in the culture medium did not change. The efficiency of overexpression of the FLAG-tagged NRK2 and its co-expression with FLAG-tagged ENTs was assessed by immunoblotting using antibodies recognizing the FLAG peptide (Figure S6). A decrease in the concentration of extracellular NAR was accompanied by an increase in the amount of Nam in the culture medium both after overexpression of NRK2 and after its co-expression with the ENT1, ENT2, or ENT4. However, statistical significance could not be established (Figure 4B, right panel). This might indicate that the mononucleotide formed from NAR by NRK2 (NAMN) is less efficiently used for NAD^+^ synthesis compared to NR. Importantly, the concentrations of extracellular NAR and Nam remained at control levels after overexpression of NRK2, as well as after co-expression of NRK2 with ENT1 when the inhibitor NBTI was added to the culture medium (Figure 4C). Thus, the overexpression of ENT1, ENT2 and, to lesser extent, ENT4 increases NAR consumption by HEK293 cells when utilization of this nucleoside in the cytosol is stimulated by overexpression of NRK2 (Figure 4D). The increase in extracellular Nam may be explained by the fact that NAR is metabolized into NAD^+^, which is then converted to Nam through NAD^+^-consuming processes (Figure 4D). Taken together, our results suggest that members of the ENT family can mediate NAR uptake into human cells.

### 2.5. Overexpression of CNT3 in HEK293 Cells Moderately Stimulates NAR Uptake

**Figure 5:**
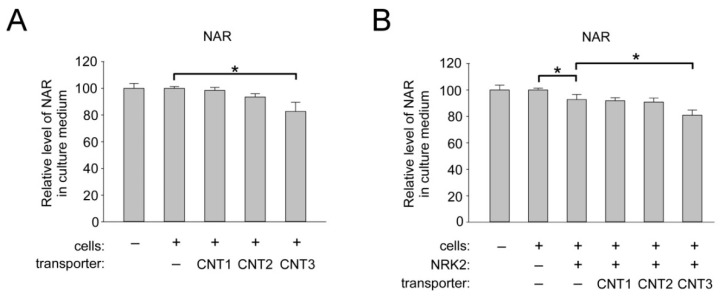
Overexpression of CNT3 moderately stimulates NAR uptake into HEK293 cells. HEK293 cells cultivated in DMEM were transiently transfected with vectors encoding FLAG-tagged CNT1, -2, or -3 or control (empty) vector (**A**). Cells were also cotransfected with a vector encoding FLAG-tagged NRK2 as indicated (**B**). Twenty-four hours after transfection, cells were treated with nicotinic acid riboside NAR (50 µM). Twenty-four hours after treatment, culture medium was analyzed by NMR spectroscopy. Relative levels of NAR in culture medium are presented. The amount of NAR in control culture media incubated without cells was taken as 100. Data are presented as mean ± S.D (*n* = 3). Statistical analysis of differences between the groups was carried out by one-way ANOVA with post hoc comparisons using Tukey test. * indicates statistical significance at *p* < 0.05.

Finally, we investigated whether members of the concentrative nucleoside transporters mediate the import of NAR into human cells. HEK293 cells were transfected with control vector or vectors encoding FLAG-tagged CNT1–3 and then incubated in the presence of NAR (50 µM). Overexpression of CNT1 and CNT2 had no effect on the efficiency of NAR consumption by HEK293 cells, whereas after overexpression of the CNT3 protein, we observed a decrease in the amount of NAR in the culture medium by 20% (Figure 5A, left panel). Note that co-expression of NRK2 with the CNTs did not change the observed effect (Figure 5A, right panel). These data demonstrate that CNT3 can facilitate NAR uptake into human cells.

## 3. Discussion

**Figure 6:**
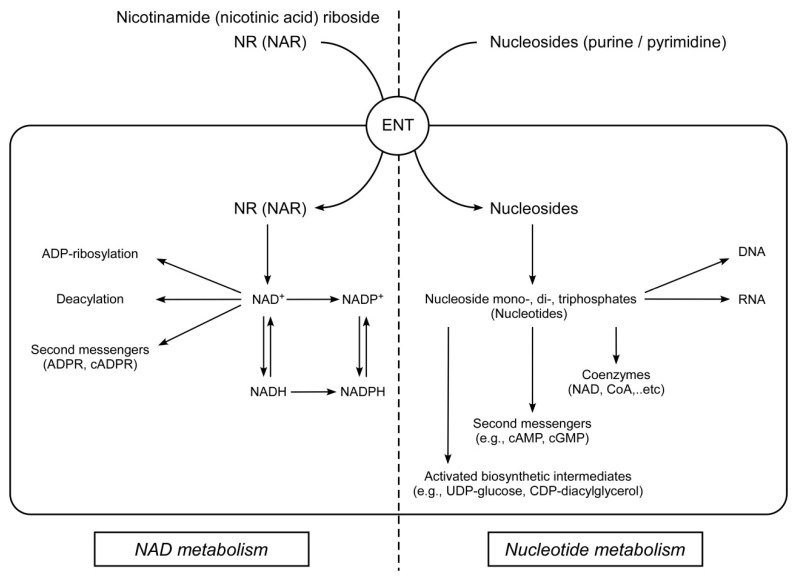
Human ENTs mediate cellular uptake of various nucleosides required for the synthesis of NAD(P)^+^ or their mono-, di-, and triphosphates needed in all branches of metabolism and signaling.

In the present study, we have confirmed at the molecular level that, indeed, members of the ENT family-proteins ENT1, ENT2, and ENT4 can import extracellular NR into cultured human cells. Moreover, we have demonstrated that the same mechanism can be used to import the deamidated form, NAR. Moreover, active import of NR and NAR into HEK293 cells occurs only under the condition of efficient utilization of nucleosides in the cytosol. Human plasma membrane carriers ENT1 and ENT2 are found in most cell types and tissues and transport a broad range of purine and pyrimidine nucleosides. ENT4 transports adenosine and monoamines in the brain and heart [35,36]. Another member of the ENT family, protein ENT3, is also widely distributed and has broad permeant selectivity, but functions predominantly intracellularly, where it is found in lysosomal and mitochondrial membranes [41,42]. ENT-dependent cellular uptake of exogenous purine and pyrimidine nucleosides is one of the major salvage pathways of nucleotide metabolism. Once in the cell, nucleosides are used to synthesize various nucleotides ((deoxy) nucleoside (di-, tri-) phosphates), which perform various vital functions in the cell (Figure 6).

Besides the fact that nucleotides are the building blocks for the synthesis of DNA and RNA, some of them are themselves important regulatory intracellular molecules. For example, energy-rich ATP and other nucleoside triphosphates control central metabolic and signaling processes. Additionally, nucleotides are found in major coenzymes such as NAD^+^, FAD, and acetyl-CoA or are used for the synthesis of second messengers such as cAMP and cGMP, as well as for the formation of chemically activated intermediates involved in the synthesis of carbohydrates and lipids such as UDP-glucose and CDP -diacylglycerol.

The fact that members of the ENT family also mediate the transport of pyridine nucleosides (NR and NAR) indicates the possibility of a functional crosstalk between the metabolisms of NAD^+^ and purine and pyrimidine nucleotides at the level of cellular import. In principle, a transient high concentration of one nucleoside, for example, during a supplementation intervention, could potentially outcompete the import of other critical nucleosides. This may lead to perturbations in nucleotide synthesis or adenosine signaling through the P1 receptors, which regulate a number of biological functions [43].

As shown in this study and a previous study [40], once NR enters the cell, it is actively metabolized to form Nam. The observed formation of Nam can be explained by the fact that NR is metabolized into NAD^+^, which is then converted to Nam by NAD^+^-consuming enzymes. It cannot be ruled out that NR or its phosphorylated form, NMN, are converted to Nam prior to their conversion to NAD^+^. These processes would run in parallel; however, their individual contributions remain to be established. Unlike NR, NAR is not actively metabolized in the cytosol; however, its phosphorylation to NAMN can be effectively stimulated by overexpressing NRK2. Under these conditions, co-expression with ENTs leads not only to a significant drop in the level of extracellular NAR, but also to an increase in the amount of Nam in the nutrient medium. The lack of measurable utilization of NAR, in contrast to NR, can explain our previous observations, according to which NAR, added to HepG2 cells in the presence of FK866, partially restored cell viability already at a concentration as low as 1 µM, whereas to achieve the same effect, a 10-fold higher NR concentration was required [33]. However, NAD^+^ turnover rates have not been measured under these conditions. Therefore, it is not possible to evaluate whether NAD^+^ metabolism is restricted to some key functions when only NAR can be used as a precursor or whether there is some futile consumption of NR.

We have recently established that both NR and NAR can be produced in cultured human cells through dephosphorylation of the corresponding mononucleotides, NMN and NAMN, by cytosolic 5′-nucleotidases. Moreover, we showed that NAR and presumably NR are excreted by cells, which thereby can provide precursors for NAD^+^ synthesis in neighboring cells [33]. Consequently, these nucleosides represent natural intermediates of human NAD^+^ metabolism. In addition, a physiologically relevant contribution of NR and NAR as NAD^+^ precursors for some cell types could enable selective facilitation of their NAD^+^ synthesis. In this regard, the question arises as to how the nucleosides are released from the producing cells. Is the export also mediated by members of the ENT family, as is the case for adenosine release [44]? Perhaps, there is another alternative mechanism of NR and NAR release that still needs to be uncovered.

## 4. Materials and Methods

### 4.1. Materials

Unless otherwise specified, all chemicals and reagents were of analytical grade and were purchased from Sigma (Saint Louis, MO, USA) and Amresco (Solon, OH, USA). Cell culture reagents were from Gibco (Waltham, MA, USA), Greiner Bio-One (Monroe, NC, USA), and Orange Scientific (Braine-l’Alleud, Belgium). HPLC-grade methanol and acetonitrile were obtained from Merck (Darmstadt, Germany). The ultrapure water was obtained from a Milli-Q Synthesis purification system (Millipore, Burlington, MA, USA). NR and NAR were synthesized as reported previously [45]. DNA-modifying and restriction enzymes were purchased from Thermo Scientific (Waltham, MA, USA). The following antibodies from Sigma (Saint Louis, MO, USA) were used: mouse anti-FLAG M2 and HRP-conjugated rabbit anti-mouse antibodies. Enhanced chemiluminescence (ECL) reagents were from GE Healthcare (Chicago, IL, USA).

### 4.2. Cell Culture

HEK293 cells (obtained from American Type Culture Collection (ATCC, Manassas, VA, USA) were cultivated in Dulbecco’s modified Eagle’s medium supplemented with 10% fetal bovine serum, 2 mM glutamine, and penicillin/streptomycin. The cells were cultured at 37 °C in a humidified atmosphere of 5% CO_2_. Transient transfection of cells was performed using Effectene reagent (Qiagen, Hilden, Germany) or the calcium phosphate precipitation method. NR (100 µM), NAR (50 or 100 µM), Urd (150 µM), NBTI (10 µM), and FK866 (2 µM) were added to the culture medium as indicated.

### 4.3. Generation of Eukaryotic Expression Vectors

For transient expression of FLAG-tagged proteins ENT1, -2, -3, -4 and CNT1, -2, -3 in human cells, their corresponding open reading frames (ORFs) were amplified from HEK293 cDNA and inserted into the pFLAG-CMV-4 plasmid (Sigma, Saint Louis, MO, USA). DNA sequences encoding ENT4, CNT1, and CNT2 were first amplified using primers without restriction sites, and then used as templates for amplification using primers containing restriction sites for cloning into the pFLAG-CMV-4 plasmid. GenBank accession numbers for ORFs and primers used are provided in the Table S1. All cloned DNA sequences were verified by DNA sequence analysis. The pFLAG-CMV-5a plasmid encoding FLAG-tagged NRK2 was described before [37].

### 4.4. Western Blotting

Cells were washed with phosphate-buffered saline (PBS) and lysed in 50 mM Tris/HCl, pH 6.8, 4% (*w/v*) SDS, 5M urea, 10% (*v/v*) glycerol, 5% β-mercaptoethanol, and 0.01% (*w/v*) bromophenol blue for 30 min at 37 °C. Gel electrophoresis and immunoblotting were carried out according to standard procedures. ECL was used for immunodetection. Pictures were taken using the ChemiDoc Imaging System (Bio-Rad, Hercules, CA, USA).

### 4.5. Metabolite Measurements

For metabolite extraction, HEK293 cells (2 × 10^7^) grown on 100 mm cell culture plates were washed twice with ice-cold PBS and put on ice. Following addition of 80% methanol, the cells were kept on ice for 30 min. Thereafter, the cells were scraped off and centrifuged at 15,000× *g* for 30 min at 4 °C. The obtained pellets were used for protein determination using BCA Protein Assay kit (Thermo Fisher Scientific, Rockford, IL, USA). Cell extracts were lyophilized and then resuspended in DBP buffer, D2O-based buffer containing 50 mM NaPi (pH 6.5), and 1 mM sucrose as a chemical shift reference (d(1H), 5.42 ppm) and internal standard for quantification. To remove oxygen, the samples were kept under vacuum (80 mm Hg) for 10 min with occasional agitation. Samples were stored at −80 °C until NMR analysis. Culture media from HEK293 cells were collected and stored at −80 °C. To precipitate proteins, the samples were incubated on ice with two volumes of acetonitrile for 30 min and then centrifuged at 15,000× *g* for 30 min at 4 °C. Supernatants were then treated in the same way as the cell extracts. All NMR experiments were performed using a Varian DirectDrive NMR System 700-MHz spectrometer equipped with a 5-mm z-gradient salt-tolerant as described in [46]. Briefly, the one-pulse sequence with the suppression of solvent signal by presaturation was used for acquisition of ^1^H spectra. The following acquisition parameters were used: relaxation delay, 2.0 s; acquisition time, 3.0 s; and number of scans, 256–1536. Data were acquired using VNMRJ 4.2 (Agilent Technologies, Santa Clara, CA, USA) and then analyzed by Mestrelab Mnova (version 12; Mestrelab, Santiago de Compostela, Spain). The concentrations of NAD^+^, NR, Nam, and NAR were determined by integration of the corresponding non overlapping proton signals with the following chemical shifts: 9.34 ppm, 9.15 ppm, 8.84 ppm, and 8.44 ppm for NAD^+^, 9.62 ppm for NR; 8.72 ppm and 7.60 ppm for Nam; 9.47 ppm for NAR. The concentration of Urd was determined by the integration of the overlapping proton signals from two proton groups (two doublets) with the chemical shifts 5.92 ppm.

### 4.6. Statistical Analysis

Statistical analysis was performed using the SigmaPlot 12.0 (Systat Software Inc., San Jose, CA USA). Differences between groups were analyzed using one-way ANOVA with Tukey’s post-hoc test. *p*-values < 0.05 were considered to be significant.

## Data Availability

The data presented in this study are available from the corresponding author upon request.

## Supplementary Materials

The following are available online at https://www.mdpi.com/1422-0067/22/3/1391/s1, Figure S1: The effect of ENT inhibition on NR utilization by HEK293 and HeLa cells for NAD^+^ biosynthesis, Figure S2: Overexpression of ENT1, ENT2, and ENT4 stimulates NR uptake into HEK293 cells, Figure S3: Overexpression of CNT1, CNT2, and CNT3 stimulates Urd uptake into HEK293 cells, Figure S4: Extracellular NAR supports NAD^+^ generation in HEK293 cells, Figure S5: The effect of overexpression of ENT1–4 and CNT1–3 on NAR uptake into HEK293 cells, Figure S6: Co-expression of NRK2 and ENT1, ENT2, ENT2, or ENT4 in HEK293 cells, Table S1: Primers used for cloning of the sequences encoding human ENT1–4 and CNT1–3 proteins into the pFLAG-CMV-4 vector.
