# Supplementary material for: Equilibrative Nucleoside Transporters Mediate the Import of Nicotinamide Riboside and Nicotinic Acid Riboside into Human Cells
Source: Int J Mol Sci. 2021 Jan 30;22(3):1391. doi: 10.3390/ijms22031391 (PMC7866510; doi:10.3390/ijms22031391)
Supplement: Supplementary file 1 [file ijms-22-01391-s001.pdf]

Supplementary data

Figure S1

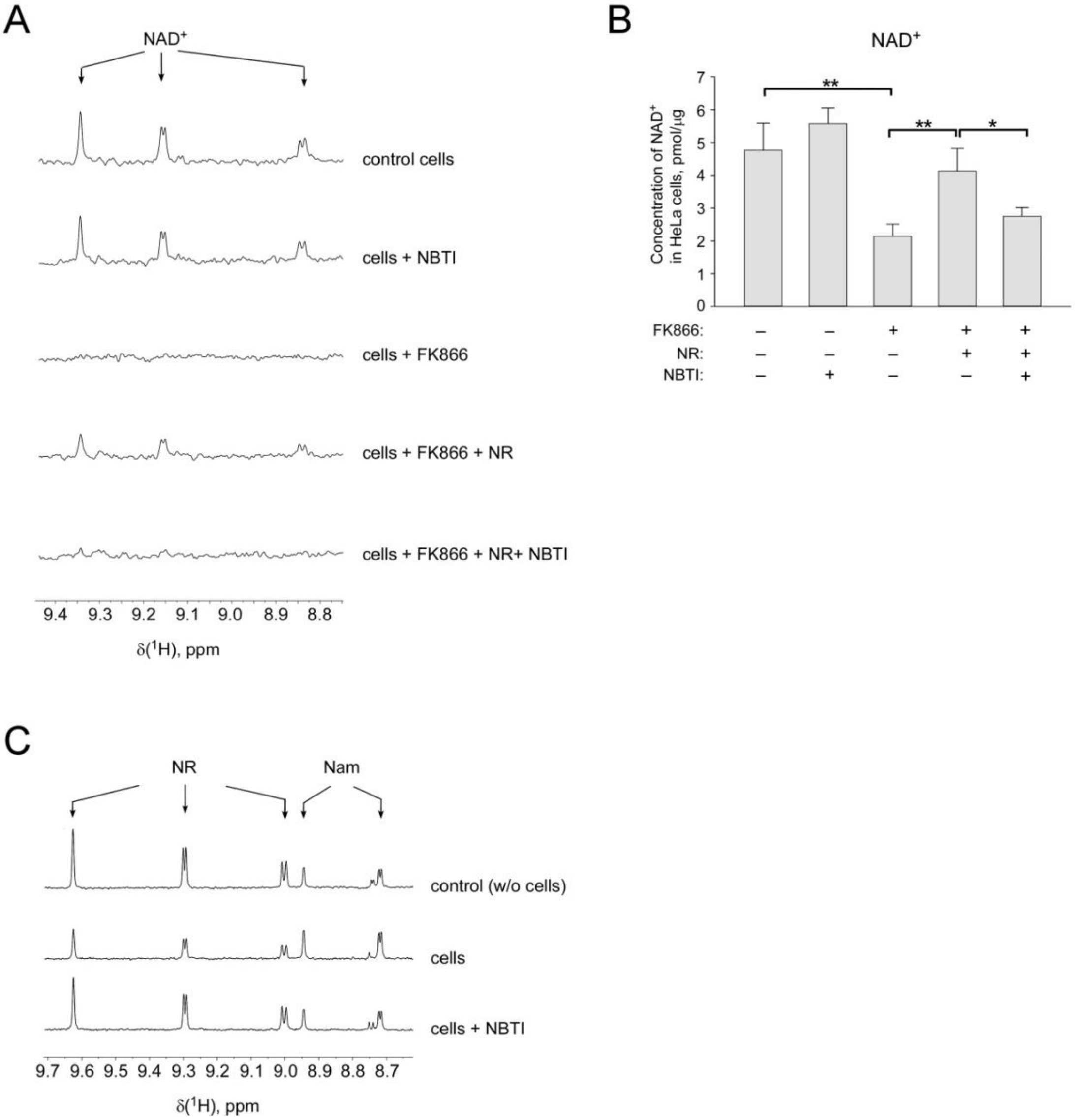

Figure S1. The effect of ENT inhibition on NR utilization by HEK293 and HeLa cells for NAD^+ biosynthesis. HEK293 (A and C) and HeLa (B) cells were cultivated in Dulbecco's modified Eagle's medium (DMEM) containing Nam, supplemented with 10% fetal bovine serum (FBS). Cells were treated with nicotinamide riboside (NR) (100 μM) and inhibitor of equilibrative nucleoside transporters S-(4-nitrobenzyl)-6-thioinosine (NBTI) as indicated. To inhibit NAD^+ synthesis from Nam, cells were also treated with FK866 (A and B). 24 h after the treatment, cell extracts (A and B) and culture media (C) were analyzed by NMR spectroscopy. (A) represents ^{1}H NMR spectra of HEK293 cell extracts. *Arrows* indicate peaks corresponding to NAD^+. (B) The concentration of intracellular NAD^+ in HeLa cells is expressed in picomoles per microgram of total protein in cell extract. Data are presented as mean ± S.D (n = 3). Statistical analysis of differences between the groups was carried out by one-way ANOVA with post hoc comparisons using Tukey test. * indicates statistical significance at $p < 0.05$, ** indicates statistical significance at $p < 0.01$. (C) represents ^{1}H NMR spectra of culture medium from HEK293 cells. *Arrows* indicate peaks corresponding to NR and Nam.

Figure S2

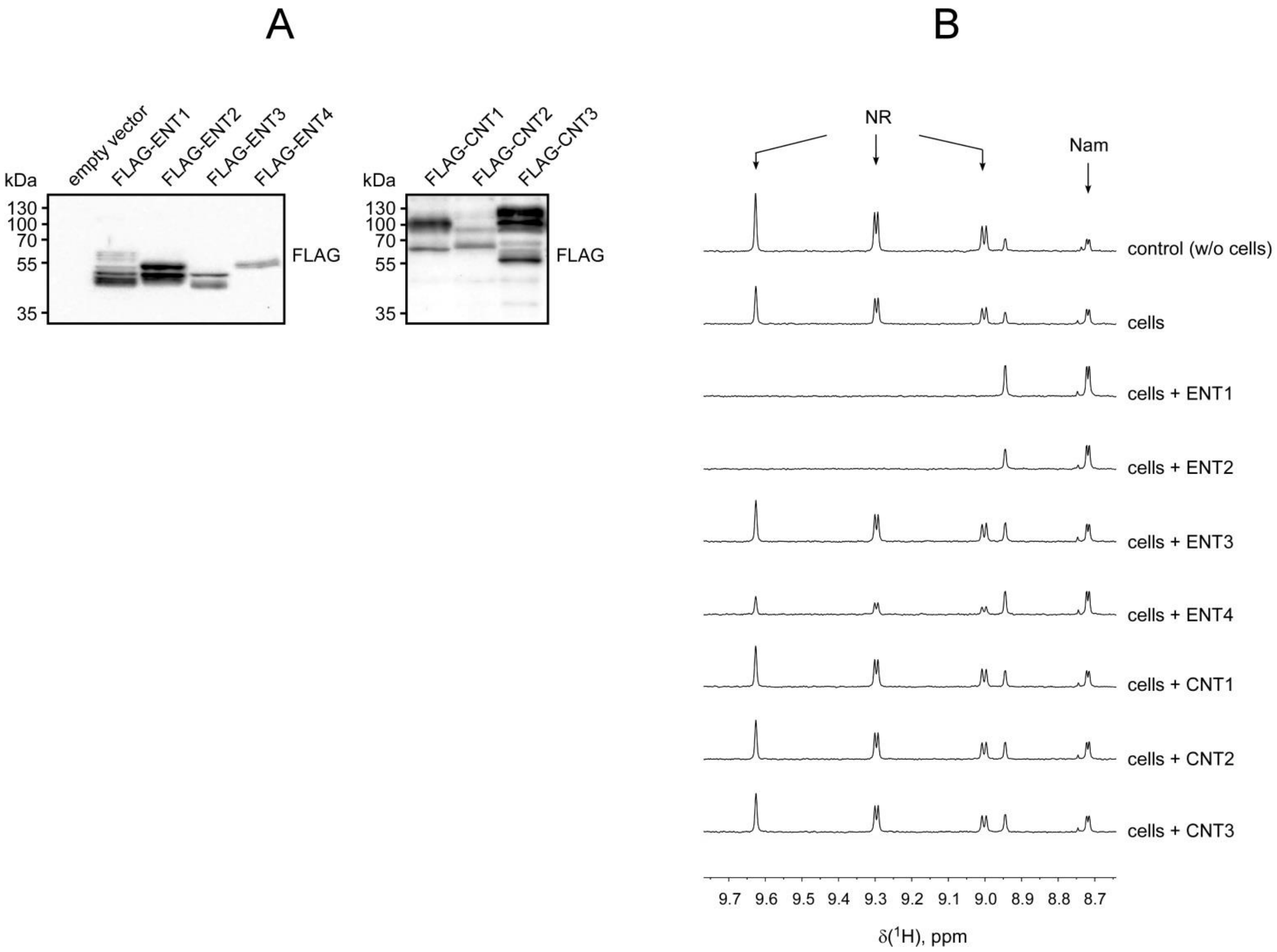

Figure S2. Overexpression of ENT1, ENT2 and ENT4 stimulates NR uptake into HEK293 cells. HEK293 cells cultivated in DMEM were transiently transfected with empty vector or with vectors encoding FLAG-tagged ENT1-4 or CNT1-3. (A) 48 hours after transfection the expression of FLAG-tagged proteins was confirmed by immunoblotting using antibody to FLAG peptide. (B) 24h after transfection cells were treated with NR (100 µM). 24h after treatment, culture medium was analyzed by NMR spectroscopy. Control medium was incubated under the same conditions without cells. ^{1}H NMR spectra of culture medium are presented. *Arrows* indicate peaks corresponding to NR and Nam.

Figure S3

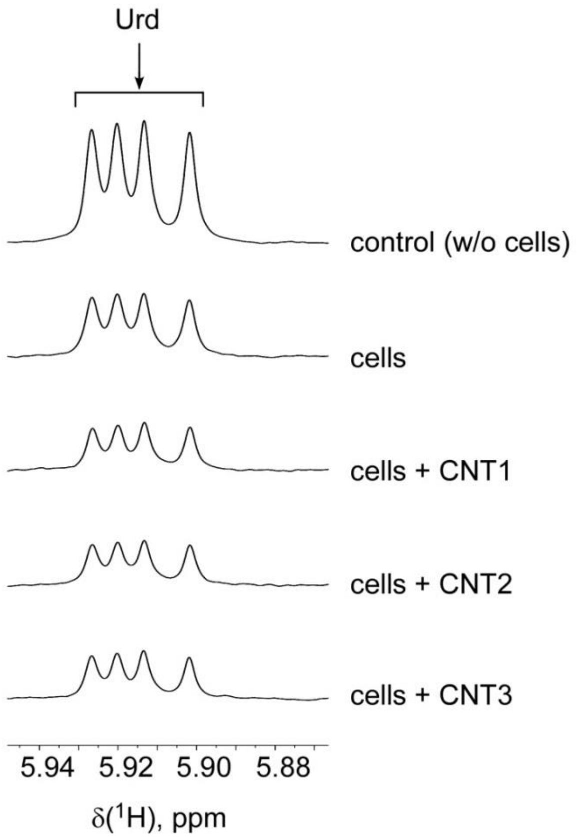

Figure S3. Overexpression of CNT1, CNT2 and CNT3 stimulates Urd uptake into HEK293 cells. HEK293 cells cultivated in DMEM were transiently transfected with empty vector or with vectors encoding FLAG-tagged CNT1, 2, or 3 as indicated. 24h after transfection cells were treated with uridine (Urd) (150 μM). 24h after treatment culture medium was analyzed by NMR spectroscopy. Control medium was incubated under the same conditions without cells. ^{1}H NMR spectra of culture medium are presented. *Arrow* indicates peaks corresponding to Urd.

Figure S4

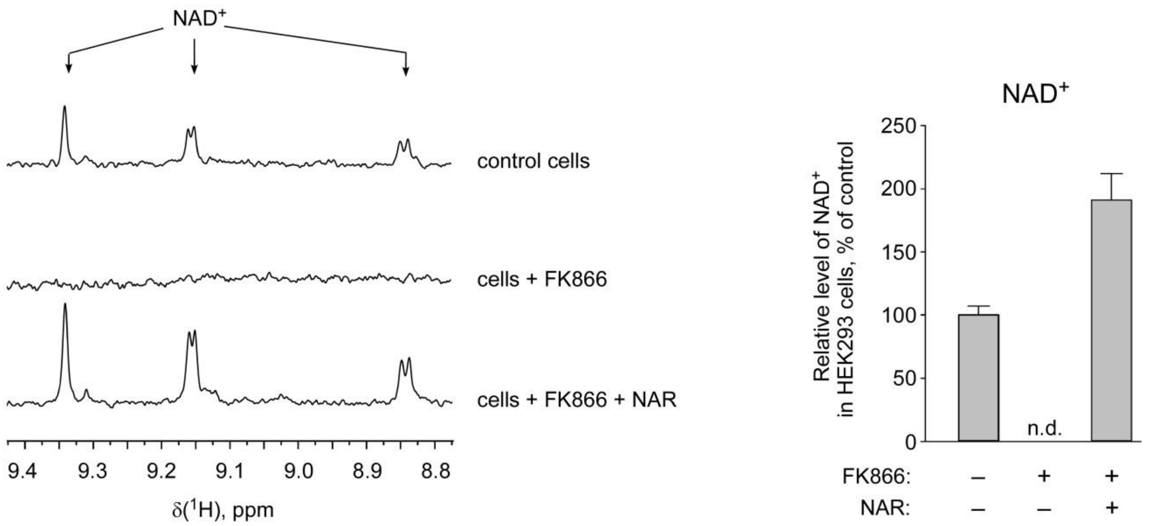

Figure S4. Extracellular NAR supports NAD$^+$ generation in HEK293 cells. HEK293 cells were cultivated in DMEM containing Nam, supplemented with 10% FBS. To inhibit NAD$^+$ synthesis from Nam, cells were treated with FK866 (2 μM). Cells were also treated with nicotinic acid riboside (NAR) at a concentration of 100 μM. 24 h after the treatment, cell extracts were analyzed by quantitative NMR spectroscopy. The left panel represents ^{1}H NMR spectra of HEK293 cell extracts. *Arrows* indicate peaks corresponding to NAD$^+$. The right panel shows relative levels of intracellular NAD$^+$ in cell extracts. Amount of NAD$^+$ in control cell extract obtained from untreated cells was taken as 100%. Data are presented as mean ± S.D. n.d. – not detected.

Figure S5

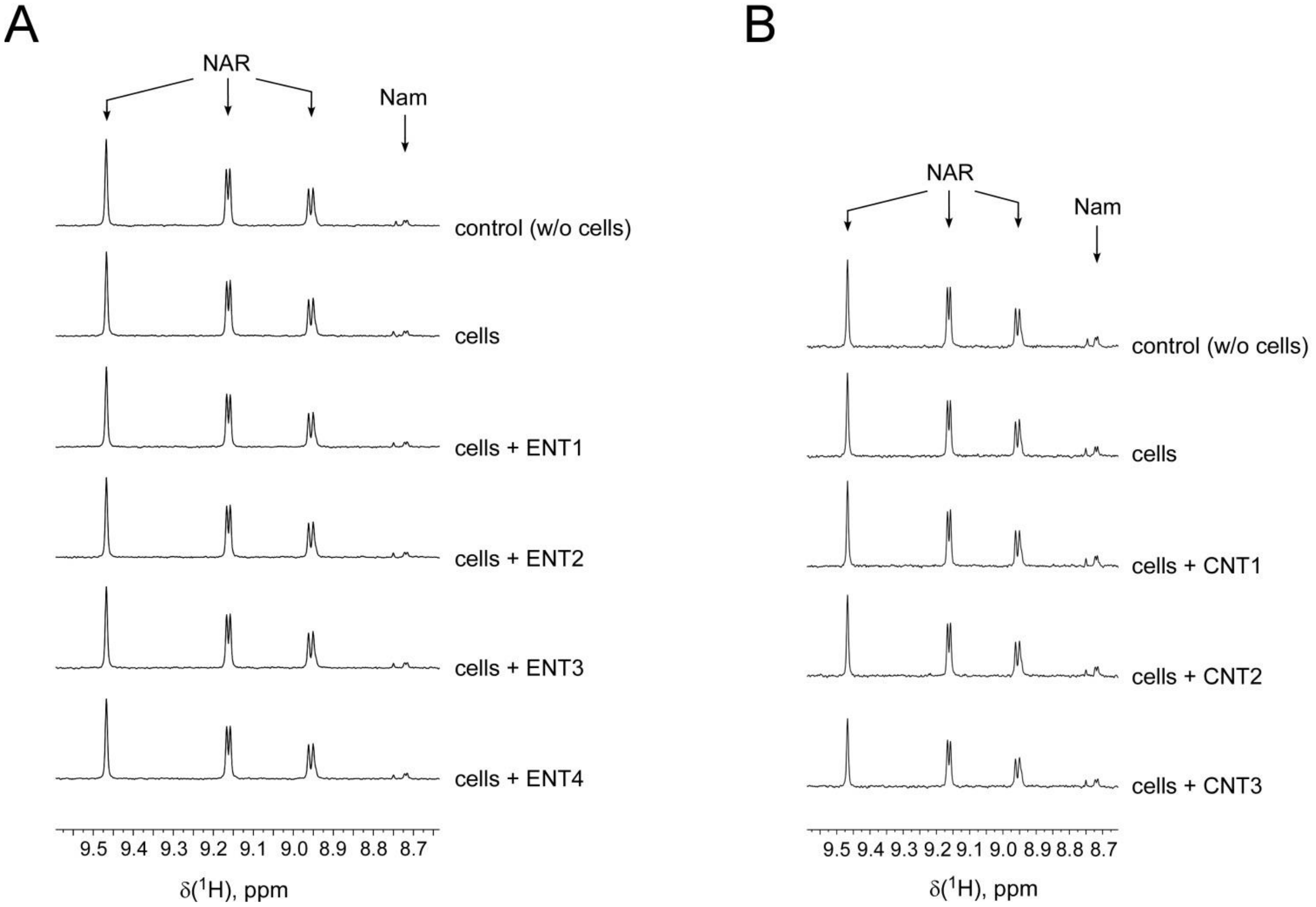

Figure S5. The effect of overexpression of ENT1-4 and CNT1-3 on NAR uptake into HEK293 cells. HEK293 cells cultivated in DMEM were transiently transfected with empty vector or with vectors encoding FLAG-tagged ENT1, 2, 3, 4 (A) or FLAG-tagged CNT1, 2, 3 (B) as indicated. 24h after transfection cells were treated with nicotinic acid riboside (NAR) at a concentration of 100 µM (A) or 50 µM (B). 24h after treatment culture medium was analyzed by NMR spectroscopy. Control medium was incubated under the same conditions without cells. ^{1}H NMR spectra of culture medium are presented. *Arrows* indicate peaks corresponding to NAR and Nam.

Figure S6

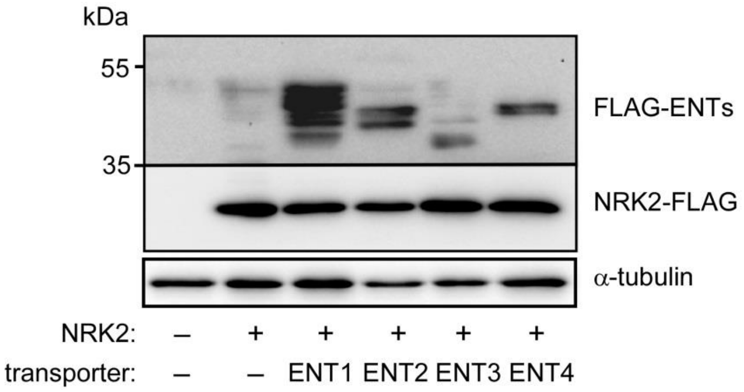

Figure S6. Co-expression of NRK2 and ENT1, ENT2, ENT2 or ENT4 in HEK293 cells. HEK293 cells cultivated in DMEM were transiently cotransfected with vector encoding FLAG-tagged NRK2 and vectors encoding FLAG-tagged ENT1, ENT2, ENT2 or ENT4 as indicated. 48 hours after transfection the expression of FLAG-tagged proteins was confirmed by immunobloting using antibody to FLAG peptide. α-tubulin served as a loading control.

Table S1. **Primers used for cloning of the sequences encoding human ENT1-4 and CNT1-3 proteins into the pFLAG-CMV-4 vector.**

Gene (GenBank accession number)		Primers	Encoded protein
SLC29A1 (NM_001078177)	Forward	CTA TAG ATC TTA CAA CCA GTC ACC AGC C	FLAG-ENT1
	Reverse	CAT TTC TAG ATC ACA CAA TTG CCC GGA AC	
SLC29A2 (NM_001300868)	Forward	CTA TAA GCT TGC GCG AGG AGA CGC CCC	FLAG-ENT2
	Reverse	CAT TTC TAG ATC AGA GCA GCG CCT TGA AGA GG	
SLC29A3 (NM_018344)	Forward	GTA TAA GCT TGC CGT TGT CTC AGA GGA C	FLAG-ENT3
	Reverse	CAT TTC TAG ACT AGA TGA GGT GCA CCA GG	
SLC29A4 (NM_001040661)	Forward (w/o restriction site)	ATG GGC TCC GTG GGG AGC CAG C	FLAG-ENT4
	Reverse (w/o restriction site)	TCA GAG GCC TGC GAG GAT GGA ACC	
	Forward	GTATAAGCTTGGCTCCGTGGGGAGCCAG	
	Reverse	CAT TTC TAG ATC AGA GGC CTG CGA GGA TGG	
SLC28A1 (NM_004213)	Forward (w/o restriction site)	ATG GAG AAC GAC CCC TCG AGA CG	FLAG-CNT1
	Reverse (w/o restriction site)	TCA CTG TGC ACA GAT CGT GTG GTT G	
	Forward	CAG ATA GAA TTC AGA GAA CGA CCC CTC GAG	
	Reverse	CAT TTC TAG ATC ACT GTG CAC AGA TCG TG	
SLC28A2 (NM_004212)	Forward (w/o restriction site)	ATG GAG AAA GCA AGT GGA AGA CAG	FLAG-CNT2
	Reverse (w/o restriction site)	TTA GGC ACA GAC GGT ATT GTT GTA G	
	Forward	GTA TAA GCT TGA GAA AGC AAG TGG AAG AC	
	Reverse	GAT ATC TAG ATT AGG CAC AGA CGG TAT TG	
SLC28A3 (NM_001199633)	Forward	CTA TAA GCT TGA GCT GAG GAG TAC AGC	FLAG-CNT3
	Reverse	GAT CAG ATC TTC AAA ATG TAT TAG AGA TCC	
